# Reciprocal relationship between expression of hypoxia inducible factor 1*α* (HIF-1*α*) and the pro-apoptotic protein Bid in *ex vivo* colorectal cancer

**DOI:** 10.1038/sj.bjc.6604474

**Published:** 2008-07-22

**Authors:** M M Seenath, D Roberts, C Cawthorne, M P Saunders, G R Armstrong, S T O'Dwyer, I J Stratford, C Dive, A G Renehan

**Affiliations:** 1 Clinical and Experimental Pharmacology, Paterson Institute of Cancer Research, Manchester, UK; 2 Department of Surgery, Christie Hospital NHS Foundation Trust, Manchester, UK; 3 Department of Clinical Oncology, Christie Hospital NHS Foundation Trust, Manchester, UK; 4 Department of Histopathology, Hope Hospital, Salford, UK; 5 School of Pharmacy and Pharmaceutical Sciences, University of Manchester, Manchester, UK; 6 School of Cancer and Imaging Sciences, University of Manchester, Manchester, UK

**Keywords:** colorectal cancer, tumour hypoxia, Bid, HIF-1*α*

## Abstract

Hypoxia inducible factor 1 (HIF-1) represses the transcription of pro-apoptotic *bid* in colorectal cancer cells *in vitro*. To assess the clinical relevance of this observation, HIF-1*α* and Bid were assessed in serial sections of 39 human colorectal adenocarcinomas by immunohistochemistry. In high HIF-1*α* nuclear-positive cell subpopulations, there was a significant reduction in Bid expression (ANOVA, *P*=0.04). Given the role of Bid in drug-induced apoptosis, these data add impetus to strategies targeting HIF-1 for therapeutic gain.

Approximately 30–50% of patients diagnosed with colorectal cancer (CRC) (35 000 new cases per year in the United Kingdom) will either present with or develop distant metastases. For these patients, management with combination chemotherapy offers survival prolongation (Saunders and Iveson, 2006), but the disease course is frequently characterised by acquired drug resistance manifesting as tumour progression and the need to alter treatment.

Tumour hypoxia and suppression of apoptosis are important drivers of drug (Makin and Dive, 2003; Shannon *et al*, 2003) and radiotherapy (Saunders *et al*, 2002) resistance in solid tumours. The transcription factor, hypoxia inducible factor-1 (HIF-1), consists of *α* and *β* subunits: the former is ubiquitinated and degraded in normoxia but stabilised in hypoxia; the latter is constitutively expressed independent of oxygen (Semenza, 2003). Under hypoxic conditions, HIF-1*α* is translocated to the cell nucleus where it dimerises with HIF-1*β* to bind hypoxia-responsive elements (HRE) in target gene promoters. Hypoxia inducible factor-1 targets encode multiple components of adaptive pathways, including increased glucose uptake, pH stabilisation (via carbonic anhydrase (CA-IX) (Wykoff *et al*, 2000), and regulation of apoptosis (Greijer and van der Wall, 2004).

Of the Bcl-2 family of apoptosis regulatory proteins, pro-apoptotic Bid is of particular interest as it links the mitochondrial (intrinsic)- and death receptor (extrinsic)-driven apoptotic pathways (Andersen *et al*, 2005; Letai, 2008). In exploring links between tumour hypoxia and apoptosis, we previously demonstrated that HIF-1 repressed transcription of *bid* in CRC cell lines *in vitro* (Erler *et al*, 2004). Moreover, a reciprocal relationship was demonstrated between hypoxic regions and Bid expression in CRC xenografted tumours (Erler *et al*, 2004). This study sought to determine the clinical significance of these observations and examined the relationship between the number of cells with stabilised nuclear HIF-1*α* and Bid expression in *ex vivo* CRC tissue. To assess HIF-1 functionality, nuclear HIF-1*α* expression was examined in parallel with CA-IX.

## Materials and methods

### Patients

Tissues from 39 patients with colorectal adenocarcinoma were used from the joint Christie Hospital and Hope Hospital CRC tissue banks (Manchester, UK) under ethical approvals (LREC-02/051; 03/SM/449). Clinical details have been published previously (Renehan *et al*, 2000; Bibi *et al*, 2006). All samples were from luminal tumour edges and processed, formalin-fixed, and paraffin-embedded using standard operational procedures.

### Immunohistochemical staining and analysis

**Figure 1:**
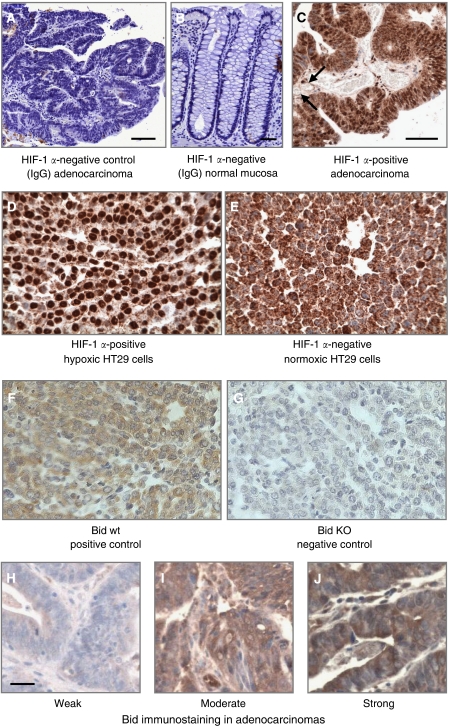
Experimental controls for immunohistochemistry. (**A**) Substitution of the primary antibody with mouse IgG1 at the same dilution was used as a negative control in all experiments. (**B**) Normal large bowel mucosa was an additional IgG1-negative control. (**C**) Representative immunostaining for HIF-1*α* demonstrating immunopositivity in close proximity to blood vessels (arrows). Both nuclear and cytoplasmic staining is seen. (**D** and **E**) Formalin-fixed and paraffin-embedded pellets of HT-29 human colorectal cancer cells previously cultured for 16 h in normoxic or hypoxic conditions (1% O_2_) and stained for HIF-1*α*. Only nuclear staining, as seen in hypoxic cells, was considered positive; we considered the diffuse cytoplasmic staining in normoxic cells as nonspecific, reasoning that HIF-1 is not transcriptionally active in cytosol. (**F** and **G**) Formalin-fixed and paraffin-embedded pellets of Bid knockout and wild-type mouse embryonic fibroblasts (kindly provided by SJ Korsmeyer, Harvard Medical School, Boston, MA, USA) were used as positive and negative controls for Bid. Pellets were processed identical to that for human tumour specimens. (**H**–**J**) Bid immunostaining in human adenocarcinomas with representative sections of weak (score 1), moderate (score 2), and strong (score 3) staining. The magnification bar for all sections is 50 *μ*m.

Antibodies used were mouse monoclonal anti-human HIF-1*α* (610958, diluted 1 : 100, BD Biosciences, Oxford, UK); goat polyclonal anti-human Bid (1 : 50, Santa Cruz Biotechnology, CA, USA); and mouse anti-human CA-IX (anti-MN 75, 1 : 20, kindly provided by Bayer Diagnostics). Sections of 4  *μ*m were stained as previously described (Wilson *et al*, 2000). Before HIF-1*α*, and Bid immunostaining, antigen retrieval was by microwaving in 10 mM citrate buffer (pH 6: 2 × 10 min), and the Tyramide Signal Amplication System (NEN Life Sciences, Boston, MA, USA) was used to optimise HIF-1*α* staining. All staining was completed using diaminobenzidene (EnVision HRP kit, Dako, Cambridge, USA) for 2–3 min before counterstaining with haematoxylin. A variety of approaches were used to control the experiment (see legend, Figure 1). Batch-to-batch variation was assessed using two regions with clear high or low nuclear HIF-1*α* expression, comparing sections from these with each batch.

Hypoxia inducible factor-1*α* expression was heterogeneous both within and between tumours. To quantify this, a scoring system was developed with nuclear HIF-1*α* expression as a continuous variable, and the number of immunopositive cells expressed per area (see below). Immunopositivity was defined by strong nuclear staining; cytoplasmic staining alone was considered negative. The mean HIF-1*α* score was based on the average of three counts. A pilot study identified that all tumours were HIF-1*α* immunopositive, but that high- and low-positive subpopulations existed using a cutoff of 20 positive cells per 40 000 *μ*m^2^ (200 × 200).

Expression scores for Bid and CAIX were semiquantitative based on stain intensity (Rhodes *et al*, 2000). Bid was assigned as weak, moderate, or strong (scored as 1, 2, or 3, respectively). For CA-IX, some tumours exhibited no staining and, therefore, the scores included zero. From these scores, a consensus of three observers was taken.

To quantify specifically the relationship between nuclear HIF-1*α* and Bid expression, a technique of paired same area serial analysis was developed. Each specimen was sectioned to produce serial slides 4 *μ*m apart. Slides were viewed and imaged using a Nikon E600 microscope (× 100 magnification). A 200 × 200 *μ*m^2^ boxed area of high HIF-1*α* staining was selected and an identical box placed on the corresponding area on the Bid slide. This procedure was repeated for low HIF-1*α* areas resulting in 78 paired analyses. A series of experiments were repeated for paired HIF-1*α* and CA-IX.

### Assessment of internal validity

Interobserver variations were assessed to confirm the validity of our analytic approaches. The number of nuclear HIF-1*α*-immunopositive cells within each boxed area was counted independently by three observers (MMS, MPS, and CD). For Bid and CA-IX, tumour images were randomised before the same observer assessed staining within the assigned boxes.

### Statistical analysis

Distributions of mean nuclear HIF-1*α* were skewed, and were therefore log-transformed. Comparisons of means used univariate and multivariate analysis of variance (ANOVA). Other immunostaining scores were treated as ordinal. Interobserver variation of continuous data (mean nuclear HIF-1*α*) was assessed using intraclass correlations: for ordinal data (Bid and CA-IX), *κ*-correlations were performed. All analyses were performed using STATA (version 9.0, College Station, TX, USA).

## Results

Immunopositivity for nuclear HIF-1*α* was present in all adenocarcinomas but was heterogeneous within tumours. Hypoxia inducible factor-1*α* staining was noted adjacent to areas of necrosis but also adjacent to blood vessels (Figure 1C). Bid immunostaining was diffuse non-nuclear and positive in all sections but its intensity varied between tumours (Figure 1E).

**Figure 2:**
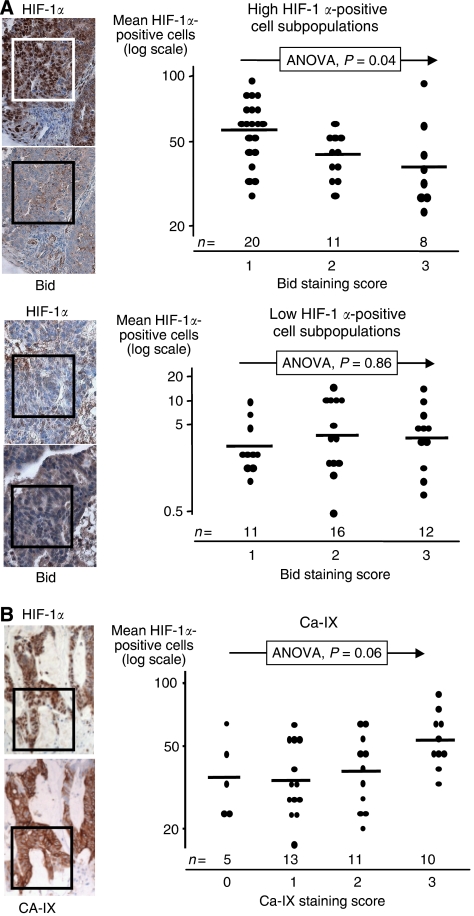
(**A**) Hypoxia inducible factor-1*α*-positive nuclei scores *vs* Bid category scores in the paired same area serial analysis. Upper panel represents high mean HIF-1*α* scores (greater than 20 positive nuclei per area). Lower panel represents low mean HIF-1*α* scores. Boxes represent 200 × 200 *μ*m^2^. Geometric means (horizontal lines) are compared across the categories using an ANOVA model. *Y* axis is log-transformed. *n* indicates number of tumours per Bid category score. (**B**) Similar graphic representation of HIF-1*α*-positive nuclei scores *vs* CA-IX expression.

Areas of high HIF-1*α* immunopositivity were often associated with low Bid expression, whereas areas of low HIF-1*α* immunopositivity were associated with variable Bid expression. This was quantified as follows: for high-HIF-1*α* areas, there was a stepwise reduction in Bid expression with increasing mean HIF-1*α* positivity (ANOVA, *P*=0.04) (Figure 2A). This relationship was absent for low HIF-1*α* areas.

Mean HIF-1*α* immunopositivity correlated with the total number of epithelial cells per square (*r*=0.86, *P*<0.001), that is, cell density. To assess this further, a multivariate ANOVA model was generated, and demonstrated the attenuation of the extent of inverse association between mean HIF-1*α* and Bid expression in the high HIF-1*α* subpopulations (*P*=0.07).

In the assessment of HIF-1 functionality, there was coincident staining of mean HIF-1*α* immunopositivity with CA-IX immunopositivity (ANOVA, *P*=0.06) (Figure 2B).

### Internal validity

The intraclass correlation for HIF-1*α* immunopositivity counts (among high HIF-1*α* areas) was 0.77 (95 confidence interval: 0.67–0.88), with an estimated reliability of 91%. The *κ*-correlations for observer 1 *vs* 2, 1 *vs* 3, and 2 *vs* 3 were as follows: Bid: 0.72, 0.72, and 0.83; CA-IX: 0.55, 0.75, and 0.56; respectively.

## Discussion

We purposefully limited our clinical study design to test a single hypothesis that HIF-1 represses *bid* transcription leading to reduced Bid expression in *ex vivo* CRC having shown that hypoxia-driven downregulation of Bid occurred via a HIF-1-dependent pathway in CRC cell lines *in vitro*. We had demonstrated *in vitro* that this mechanism was operational during hypoxic resistance to etoposide and oxaliplatin, which was overcome to a degree by forced maintained expression of Bid. When CRC lines were xenografted into CD1 nude mice, a striking reciprocal relationship was observed between the hypoxia marker pimonidazole, which formed a penumbra adjacent to necrotic regions in tumours (a location typically associated with chronic hypoxia), and Bid expression, which stained more positively away from the necrotic regions (Erler *et al*, 2004). However, although the tumour–xenograft model is widely used for preclinical testing of anticancer agents, it has two key limitations – first, the degree of genetic heterogeneity observed in most human tumours does not exist in xenografts; and second, the native stromal microenvironment is lacking. Thus, it was necessary to test the HIF-1*α* and Bid relationship in human tissues.

The human data that emerged were consistent with HIF-1*α*-mediated repression of Bid occurring in human CRC. However, this relationship was modest, and attenuated after the adjustment for cell density. It is not surprising that HIF-1*α* expression varied after this adjustment, as its induction may be influenced by hypoxic and non-hypoxic mechanisms (Semenza, 2003). Hence, for example, in the human specimens, HIF-1*α* immunostaining was observed adjacent to oxygen-rich intratumoral blood vessels. One may expect the relationship between transcriptionally active HIF-1 and Bid to be independent of these mechanisms, but other regulators of Bid expression (e.g., p53 loss) (Sax *et al*, 2002) may also be operational in CRC tissue. We speculate that the absence of a relationship in low-score HIF-1*α* may indicate a threshold level for *bid* transcriptional repression to predominate.

As an additional dimension of complexity, it is increasingly clear that modulation of tumour apoptosis may occur through HIF-1-independent pathways, for example, Bad activation and cell survival through an adenosine–Akt pathway in glioblastoma cells (Merighi *et al*, 2007).

Strengths of our study were as follows: first, we used robust staining controls; second, we determined HIF-1*α* immunopositivity as a continuous variable allowing analyses that included adjustments for cell density; third, we developed and validated the use of the paired same area serial analysis; and fourth, we assessed HIF-1*α* functionality in the CRC tissues by demonstrating coincident expression with CA-IX, an established HIF-1 transcriptional target.

Tumour hypoxia offers a selective approach to the treatment of cancer, as, with only few exceptions, normal tissues do not usually experience low oxygen tension. Accordingly, HIF-1 is considered an attractive anticancer drug target (Semenza, 2003; Welsh and Powis, 2003; Melillo, 2007). Hypoxia inducible factor-1 inhibition could potentially ablate several adaptive responses that allow tumour cell survival under conditions of compromised oxidative phosphorylation. Negating HIF-1 functions improves response to therapy (Hussein *et al*, 2006) and small-molecule HIF-1 inhibitors are currently in early clinical trial (Semenza, 2003; Melillo, 2007). The data presented here give additional impetus to targeting HIF-1 for therapeutic gain, as it is predicted to increase Bid levels and, in turn, reduce the threshold for apoptosis.
